# Shattered Safety? Developmental Trajectories for Conspiracy Mentality and Violent Extremist Attitudes

**DOI:** 10.1007/s10964-026-02350-9

**Published:** 2026-04-29

**Authors:** Hanne M. Duindam, Lucia Hernandez-Pena, Jessica J. Asscher, Denis Ribeaud, Manuel Eisner

**Affiliations:** 1 Clinical Child, Family and Education Studies, Faculty of Social Sciences, Utrecht University, Heidelberglaan 1, Utrecht, 3582 CE the Netherlands; 2 Darwin College, University of Cambridge, Silver Str, Cambridge, CB3 9EU UK; 3 Department of Psychiatry, Psychotherapy and Psychosomatics, Faculty of Medicine, RWTH Aachen, Pauwelsstrasse 30, 52074 Aachen, Germany; 4 Department of Psychology, University of Cambridge, Downing, Cambridge, CB2 3EB PI UK; 5 Jacobs Centre for Productive Youth Development, Universität Zürich, Andreasstrasse 15, Zurich, CH-8050 Switzerland; 6 Institute of Criminology, University of Cambridge, Sidgwick Ave, Cambridge, CB3 9DA UK

**Keywords:** Conspiracy mentality, Violent extremist attitudes, Adolescence, Bullying victimization, Emotional distress, General distrust

## Abstract

Violent extremism in the context of conspiracy belief poses growing societal challenges, yet developmental pathways remain poorly understood. Shattered safety—a developmental state defined by bullying victimization, emotional distress, and distrust—may lead adolescents to view the world as unsafe and unfair, heightening susceptibility to conspiracy and extremist narratives. In this study, Growth Mixture Models were conducted to identify different trajectories of shattered safety throughout adolescence, as potential developmental pathway for conspiracy mentality and violent extremist attitudes in young adulthood. Participants were 1,482 adolescents from the ongoing longitudinal Zurich Project on Social Development from Childhood to Adulthood (~ 50% female). Data from five waves were included (ages: 13, 15, 17, 20, and 24-years-old). Results revealed three developmental trajectory groups. A small group of participants (3%) experienced shattered safety throughout adolescence, reflected in increasing levels of bullying victimization, emotional distress and distrust, and this related to higher conspiracy mentality and violent extremist attitudes at age 24. Overall, these findings illustrate the importance of considering developmental factors to safeguard against harmful narratives, calling for a public health-oriented approach that promotes adolescent safety and wellbeing.

**Supplementary Information:**

The online version contains supplementary material available at 10.1007/s10964-026-02350-9.

## Introduction

Belief in conspiracy narratives has been coined a threat to security in democracies (AIVD, 2023). While most believers are not prone to violence, infamous instances of conspiracy-related violent extremism are widely discussed (Moskalenko & McCauley, 2021). Adolescence is an important period to consider for the development of conspiracy and violent extremist narratives, as views about the self and world mature during this life stage (Crocetti et al., 2021). However, little research has been conducted on developmental pathways. *Shattered safety* —a state in which adolescents’ basic beliefs about the world’s meaningfulness, benevolence, and their own worthiness are disrupted—may play a role in the development of conspirational and violent extremist attitudes (Shattered Assumption Theory; Janoff-Bulman, 1992). *Shattered safety* is conceptualized as a multidimensional state with adverse interpersonal experiences, emotional distress, and generalized distrust as its key correlates. In such a state, adolescents may become more drawn to narratives that portray the world as threatening and deceitful (conspiracy narratives), as well as to those that frame violence as a justified response to perceived malevolence (violent extremist narratives). The present study hypothesized that shattered safety may constitute a developmental pathway towards conspiracy mentality and violent extremist attitudes. The first study aim was to identify trajectories of shattered safety throughout adolescence and describe differences in sociodemographic characteristics across trajectory groups. The second study aim was to examine trajectory groups’ association with a generic tendency to believe in conspiracy narratives (conspiracy mentality; Bruder et al., 2013) and violent extremist attitudes (i.e., support or advocacy for the use of violence for social, political, religious or ideological means; Nivette et al., 2022) at age 24. Based on previous research, multiple trajectory groups of shattered safety were hypothesized, with most youth belonging to a stable low trajectory group and few to belong to more problematic increasing trajectory group(s) (Bravo et al., 2024; Shore et al., 2018). In addition, higher levels of conspiracy mentality and violent extremist attitudes at age 24 were expected to be present in participants with more problematic and increasing shattered safety trajectories throughout adolescence.

### A Theoretical Framework: Shattered Assumption Theory

The Shattered Assumption Theory (Janoff-Bulman, 1992) posits that people hold a basic set of core beliefs about themselves and the world – similar to what Bowlby referred to as “working models” – that help them interpret events, guide interactions, and function effectively. These beliefs rest on three core safety assumptions: the world is meaningful (events are just, positive things happen to good people), the world is benevolent (others are essentially trustworthy and fair), and the self is worthy (good people, including oneself, deserve positive outcomes; Janoff-Bulman, 1992). These core assumptions can be disrupted when adverse and traumatic experiences happen and co-occur with distressing and distrustful feelings and perceptions, reflecting a sense of shattered safety.

*Shattered safety* in adolescence might have an array of negative long- or short-term internalizing (depression, post-traumatic stress disorder) and/or externalizing (anti-social behavior) consequences, consistent with the principle of multifinality (i.e., same risk factors leading to multiple different outcomes; Cicchetti & Toth, 2009). Conspiracy mentality and violent extremist attitudes may represent two such outcomes, with the combination of adverse interpersonal experiences (bullying victimization), internal emotional distress, and a distrustful worldview, as one potential developmental pathway of shattered safety. Becoming a victim of bullying, can provide “evidence” for the ill intent of others, which, combined with feeling emotionally distressed and a general sense of distrust, may increase the need for meaning making and “corrective” violent action; that is something conspiracy and violent extremist narratives neatly tap into (Agnew, 2010; Bilewicz, 2022; Douglas et al., 2017).

### The Formative Period of Adolescence

Adolescence is an important phase for the maturation of views about self and the world (Crocetti et al., 2021; Rekker et al., 2015). While facing cognitive, physical, and social changes, the adolescent’s main task is to form an identity by calibrating, choosing, and ultimately forming more stable and defined attitudes about a wide domain of topics (Crocetti et al., 2018). Attitudes in the political and intergroup domain become increasingly more stable throughout adolescence (Rekker et al., 2015). Not surprisingly, during this important phase of worldview formation, there is an increased susceptibility to violent extremist and conspirational narratives. This is often attributed to adolescents’ search for recognition and belonging, increased tendency for provocation, and a draw toward adventure, making extremist milieus more appealing (Schröder et al., 2022). Accordingly, research demonstrates that the majority of offenders become radicalized before the age of 30 (Beelmann, 2020). In terms of conspiracy mentality, adolescents’ heightened proneness might be related to their cognitive stage of development, as their analytical skills – which buffer against such beliefs – are still maturing (Kriega, 2022). Moreover, young people’s long hours online, frequent exposure to misinformation (e.g., 61% in last month JIM, 2024), and lack of digital skills, are also likely contributors for adolescents’ proneness to conspiracy narratives.

### Shattered Safety in Adolescence: Pathways to conspiracy mentality and violent extremist attitudes

Even though the effects of shattered safety correlates during adolescence have not directly been studied in relation to developing a conspiracy mentality and violent extremist attitudes in adulthood (Hornsey et al., 2022; Wolfowicz et al., 2021), several models underscore the important role that adverse experiences/victimhood, emotional distress, and distrust can play. With regards to conspiracy mentality, the central role of adverse experiences/victimhood is highlighted by Bilewicz and colleagues (2022). They have proposed that the tendency to believe in conspiracy narratives can result from historical trauma, in particular feelings of powerlessness, devaluation, and an overall sense of victimhood. The key role of emotional distress and general distrust is illustrated by Van Prooijen’s Existential Threat Model, which centralizes the role of feelings of threat, anxiety, uncertainty, and distrust in heightening the appeal of conspiracy narratives (Van Prooijen, 2020). Furthermore, Douglas and colleagues’ prominent psychological needs-based framework more generally proposes that conspiracy narratives might tap into individuals’ need to feel more certain, safe, and in control (Douglas et al., 2017).

The selected shattered safety correlates of adverse experiences, emotional distress and general distrust have also been highlighted in criminological models that have proposed that violent extremist attitudes develop as a way to cope with pervasive developmental adversity or uncertainty (i.e., General Strain Theory; Agnew, 2010; Nivette et al., 2015, 2017, 2022; Simi et al., 2016). Negative experiences or treatment, which create strain, can trigger feelings of extreme anxiety, depression, or anger making one vulnerable to extreme ideologies. In an attempt to alleviate distress and restore one’s perceived position, people might turn to harmful “corrective” actions, making violent extremist attitudes more likely (Agnew, 2010; Trip et al., 2019). Especially when there is general distrust and ties to others are weak, children can feel disconnected from society, institutions and authority, creating fertile ground for violent extremist attitudes to flourish in line with Social Control Theory (Doosje et al., 2013; Gottfredson & Hirschi, 1990).

### Empirical Evidence on Shattered Safety’s Developmental Correlates

Overall, the above theories highlight how disruptions in safety, trust, and wellbeing throughout adolescence could foster the appeal of conspiracy and violent extremist narratives. Turning to empirical evidence, however, there are significant research gaps, particularly on the development of conspiracy mentality, as hardly any research has been conducted over the lifespan (Hornsey et al., 2022). For violent extremism attitudes, the developmental base is more established (e.g., Nivette et al., 2017), although there is an over-reliance on cross-sectional empirical evidence in the violent extremism field as well (Duindam et al. 2025a, b). Below an overview will be given of the current empirical evidence base for the developmental correlates of shattered safety– bullying victimization, emotional distress, distrust – and their association with conspiracy mentality and violent extremist attitudes.

#### Bullying victimization 

*Bullying victimization* is a form of developmental adversity, involving childhood maltreatment by peers, which can contribute to shattered safety and may have long-term consequences (e.g., Lereya et al., 2015). Experiencing extensive bullying can facilitate the development of a sense of victimhood, that might make one more vigilant and focused on the early identification of threat, while being less trustful of others (Bilewicz, 2022). This victim mind-set has been found to facilitate the tendency to believe in conspiracy narratives in adults (Jolley & Lantian, 2022). However, causal evidence for victimhood leading to conspiracy mentality is lacking (Imhoff et al., 2024).

With regards to violent extremism, bullying victimization as severe type of interpersonal adversity, is also considered a risk factor (Agnew, 2010). The relationship between bullying victimization and violent extremism likely comes from increased psychological vulnerabilities and a stronger desire for violent or ‘corrective” actions (Agnew, 2010, 2011; Harpviken, 2020). Moreover, observing behavior of others, including negative behaviors such as being bullied, teaches you about the acceptance and normality of that behavior (Social Learning Theory; Akers, 2009). In a systematic review on school shootings, it was found that the majority of perpetrators experienced some form of peer rejection, including verbal or physical bullying (Sommer et al., 2014). In addition, youth in Scandinavia named bullying victimization as an important reason for joining a Neo-Nazi extremist organizations (Bjørgo, 2005; Kimmel, 2007). More generally, experiences of developmental adversity and emotional consequences are often cited by former violent extremist from diverse ideological background as having played a significant role in their radicalization (Logan et al., 2024; Simi et al., 2016; Windisch et al., 2022). On the contrary, a recent systematic review did not find adverse childhood experiences to play a role in later involvement in extremist groups (Matei, 2023) and few studies have examined this link systematically or longitudinally (Grimbergen & Fassaert, 2022).

#### Emotional distress 

In general, the tendency to believe in conspiracy narratives appears higher among those who feel *emotionally distressed* (Levinsson et al., 2021) and several studies have demonstrated that conspiracy belief is associated with anxiety (e.g., Bowes et al., 2021; Grzesiak-Feldman, 2013). Emotional distress is unpleasant and could make one want to project outward in an attempt to regain control. Conspiracy narratives provide a narrative that facilitate this process (De Coninck et al., 2021). However, the direction of the effect between emotional distress and conspiracy belief is unclear, and one longitudinal study found that they can reinforce each other (Liekefett et al., 2023).

With regards to violent extremism, emotional distress could make violent extremist narratives more appealing. These ideas can promote agency and empowerment and provide purpose and meaning, even if linked to criminal behavior (Bhui et al., 2016). Conformingly, a mediating (Rousseau et al., 2019) and direct effect between violent extremism and emotional distress, in the form of depression and anxiety, has been found (Bhui et al., 2016, 2020). Contradictingly, a recent systematic review on the relationship between radicalization, violent extremism and psychiatric disorders was inconclusive (Trimbur et al., 2021).

#### Distrust 

A *general sense of distrust* seems deeply rooted in those who believe in conspiracy narratives and endorse violent extremist attitudes. In terms of conspiracy mentality, several studies have demonstrated a relation with distrust (Brotherton et al., 2013; Freeman et al., 2020; Martinez et al., 2022). Distrust has also been identified the common core of conspiracy’s mentality, as shared with populism (Thielmann & Hilbig, 2023). For violent extremist attitudes, individuals who are generally distrustful of others are expected to be less imbedded in their relations in society (Nivette et al., 2017). This could weaken the impact of society’s norms, laws and institutions on restraining deviant behavior, thereby increasing the risk for violent extremism (Becker, 2021; Holt et al., 2018). Distrust is seen as an essential contributor to societal discontent (Bos, 2023), whereas trust in various forms has been found to be a small buffer against radicalization and violent extremism (Duindam et al. , b). Research has also demonstrated that higher levels of trust in other children can play a buffering role in the development course of aggression (Malti et al., 2013).

#### Towards an integrated and longitudinal approach: Addressing the research gaps 

Overall, the prior research discussed demonstrates that bullying victimization, emotional distress, and distrust have each been associated with conspiracy mentality and violent extremist attitudes. However, this research has mostly been conducted among adults, relied on cross-sectional research designs, and has rarely examined these shattered correlates in combination – which is needed to understand outcomes from a developmental perspective (Beelmann, 2020). In the current study, these gaps are addressed by examining how shattered safety correlates jointly form a developmental pathway toward conspiracy mentality and violent extremist attitudes. Recent evidence indicates that the negative long-term impact of bullying victimization is partially mediated by distrust, suggesting that this type of victimhood, combined with distrust in particular, can erode feelings of safety and increase the chance for negative outcomes (Tsomokos & Slavich, 2024). By employing a longitudinal design, and a focus on adolescence, this research adds to the literature by identifying potential risk trajectories during a transformative developmental period marked by vulnerability towards conspiracy and extremist narratives.

## Current Study

Little developmental research has examined how conspiracy mentality emerges over time, while studies on violent extremist attitudes have also largely relied on cross-sectional data. The role of *shattered safety* correlates—adverse experiences, emotional distress, and distrust—remain unexplored in longitudinal frameworks, despite their theoretical grounding and demonstrated relevance in adult populations. The current study addresses these gaps by examining one potential developmental pathway toward conspiracy mentality and violent extremist attitudes during adolescence, an important period characterized by heightened vulnerability and worldview formation. First, shattered safety trajectories – as depicted by its developmental correlates bullying victimization, emotional distress, and distrust – were estimated over five waves during adolescence (ages: 13, 15, 17, 20, 24 years-old). Next, the different trajectory groups were associated with conspiracy mentality and violent extremist attitudes in early adulthood (24 years-old) and differences between trajectory groups in terms of sociodemographic factors (sex, SES, education, immigration background) were examined. Overall, identifying subgroups with more problematic trajectories, such as increasing shattered safety, and their association with distal outcomes, can offer insights into etiology and aid personalized prevention efforts. Significant heterogeneity in young people’s shattered safety is expected, in terms of varying trends and levels of bullying victimization, emotional distress, and general distrust, which will reveal distinct trajectory groups. Based on previous research, it is expected most youth to follow a stable low trajectory, with only a minority showing problematic increasing trajectories associated with elevated conspiracy mentality and violent extremist attitudes at age 24.

## Methods

### Participants

Participants in the present study were from the ongoing longitudinal Zurich Project on Social Development from Childhood to Adulthood (z-proso; Ribeaud et al., 2022; z proso Project Team, 2024). The z-proso study has been tracking a target sample of youth (*N* = 1675, ~ 50% female) from a target population of 2520 firth graders across 56 public elementary schools in Zurich (Switzerland), which were stratified by district and school size. Zurich is Switzerland’s largest city with approximately 430.000 residents in the city currently. It is diverse culturally and socio-economically. It is an affluent city, albeit with similarity in problems compared to other larger European/Western cities, as the study was commissioned because of an increase in youth violence in some Zurich neighborhoods. The z-proso sample is mostly representative of the city of Zurich’s youth population. Zurich has a large presence of immigrants. Therefore, more than half of the sample consists of young people with migration backgrounds. There is also a slight over-representation of those with lower socio-economic backgrounds. Overall, the diversity of the z-proso sample is representative of other larger multicultural European cities. Youth entered the study in 2004 at the age of seven and have been followed over nine waves (for a detailed cohort and study description, see Ribeaud et al., 2022).

The present study used data from wave five (13-year-olds) until the recent wave nine (24-year-olds), for which data on developmental factors were available. Details about non-response and attrition rates throughout the study are described by N. Eisner and colleagues (2019). From age 13 onward youth independently gave legal consent for study participation (Art. 16 Swiss Civil Code). Parents also received a letter about their child’s participation and if they did not agree they could withdraw consent. For wave five, unlike previous waves, all youth at baseline were allowed to be re-invited for study participation, resulting in a higher response total compared to earlier waves (Eisner et al., 2019).

Wave-wise participation rates of the target sample (*N* = 1675) were 81.5% at wave five (*n* = 1365), 86.3% at wave six (*n* = 1446), 77.9% at wave seven (*n* = 1305), 70.4% at wave eight (*n* = 1180), and 69.3% at wave nine (*n* = 1160). In the present study, youth participants were included when they had data on developmental factors for at least one wave (*n* = 1482; 88.5% of the target sample). The sample compromises 51.8% males. At wave nine (age 24), 54.4% of the sample has completed some form of higher education (36.8% at wave eight, age 20), such as university or technical school. The majority of the sample is employed at this age, with 83.0% reportedly working full-time or part-time (69.5% at wave eight).

Socioeconomic status was indicated by the International Socioeconomic Index of occupational status (ISEI) (z proso Project Team, 2024), with low scores representing e.g. unskilled work and higher scores reflecting higher status jobs (e.g. judges or medical doctors; Ganzeboom, de Graaf, & Treiman, 1992 ). The mean score was 45.97 (S.E = 0.54; ranges 16–90).

In terms of immigrant background, the sample consisted of 23.6% participants with both parents born in Switzerland, 26.9% with one parent born in Switzerland, and 49.6% with both parents born abroad, representing a very high proportion of participants with migration backgrounds (Ribeaud et al., 2022).

At wave nine (age 24), 77.5% of participants completed the conspiracy mentality scale (*n* = 1148) and 78.1% the violent extremist attitudes scale (*n* = 1157) at wave nine. Overall, attrition was higher among participants with more problem behavior, a migration background, and low educational levels (Eisner et al., 2019; Ribeaud et al., 2022). Information on participant drop-in and out in the longitudinal z-proso project are described by Eisner et al. (2019), with further cohort details reported by Ribeaud et al. (2022).

### Procedure

Ethical approval for the z-proso study was requested and received in accordance with Swiss regulations by the Ethics Committee of the Faculty of Arts and Social Sciences at the University of Zurich. The z-proso project is a multi-informant study combining teacher, parent, and youth data, in addition to observational and behavioral measures, biosampling, functional imaging and ecological momentary assessment (for more information, see Ribeaud et al., 2022; z proso Project Team, 2024). In the present study, only self-reported youth questionnaire data are used. Data-collection procedures differed slightly per wave, relevant for the present study are waves five until seven during which trained field workers administered the German paper questionnaires in classrooms after school hours. From wave eight (age 20) onward, participants were invited to a social science research laboratory in Zurich to complete computer-assisted interviews (Ribeaud et al., 2022). Youth received monetary incentives for participation, that is 30 CHF in wave five, 50 CHF in wave six, 60 CHF in in wave seven, 75 CHF in wave eight, 150 CHF in wave nine (or 100 for the online assessments).

### Instruments

For each scale, a mean score of the items was calculated as a total score, with a higher score reflecting a higher presence of the measured construct (for more information on instruments used in the z proso study, see Table 1 in the supplementary materials and the z-proso handbook; z proso Project Team, 2024). Omega's , means, standard deviations, and correlations of all constructs across waves are reported in Table 1.

#### Sociodemographic Factors 

Sociodemographic factors included sex (male/female), education (completion of higher education in wave nine), immigrant background (one or more parents born outside of Switzerland versus both parents born in Switzerland), and socioeconomic status (SES). SES was based on the International Socioeconomic Index of occupational status (ISEI) (with lower scores representing unskilled work; higher scores reflecting high status jobs; z proso Project Team, 2024).

#### Bullying Victimization 

Bullying victimization, reflecting the frequency of experiences of peer victimization in the last 12 months (i.e., “In the last 12 months, have you been…”), was measured by four items (adapted from Olweus et al., 1993) on a scale from 1 (*never*) to 6 (*almost daily*) (Murray et al., 2021). Example items include (“Have you been…”): “…purposely ignored or excluded”, “…laughed at”, and “…mocked or insulted?”. In wave nine, additional items were administered to assess victimization in young adulthood. One of the original items was discontinued at wave nine as it no longer appeared age-appropriate. The mean score was then calculated using the remaining three items.

#### Emotional Distress 

Emotional distress was measured with eight items from the Social Behavior Questionnaire (SBQ) that reflected internalizing symptoms such as anxiety (e.g., “I was anxious for no reason”) and depressive like states (e.g., “I felt unhappy”) over the past month on a scale from 1 (*never*) to 5 (*very often*; adapted from Tremblay et al., 1991, see Murray et al., 2019 for adolescent version, see ‘anxiety and depression’; z proso Project Team, 2024).

#### General Distrust 

General distrust was assessed by the Generalized Trust Scale, reverse coding all three items originally adapted from World Values Surveys (Inglehart et al., 2018). Participants indicated how true these statements (e.g., “Most people can be trusted”) were for them on 4-point scale answer format ranging from 1 (*fully untrue*) to 4 (*fully true*). All items were reverse scored to reflect a general sense of distrust.

#### Conspiracy Mentality 

Conspiracy mentality, meaning the tendency to believe in conspiracy narratives, was assessed (five items) in wave nine by an adaption of the Conspiracy Mentality Scale (Bruder et al., 2013). Items (e.g., “There are secret organizations that greatly influence political decisions”) were answered on a 4-point scale, ranging from 1 (*fully untrue*) to 4 (*fully true*).

#### Violent Extremist Attitudes 

Violent extremist attitudes, that is the support for the use of violence for political, ideological, religious, social, or economic aims was measured using four items developed by the z-proso project team (Nivette et al., 2017). There was asked to what extent they agreed with statements (e.g., “It’s sometimes necessary to use violence to fight against things that are very unjust”) on a 4-point scale from 1 (*fully untrue*) to 4 (*fully true*).

### Analysis

All analyses were conducted in *MPlus version 8.8*, including descriptives and correlational analyses as a first step. Developmental risk factors and distal outcomes were converted to z-scores to facilitate comparison.

The full-information maximum likelihood estimator with robust standard errors (MLR) was used to address missing data, thereby accommodating to non-normality and non-independence. To examine change trajectories of the shattered safety correlates of bullying victimization, emotional distress, and general distrust, a Growth Mixture Model (GMM) was conducted. This approach is well-suited for capturing the diversity in development by determining the average change in risk over time and identifying subgroups with differing patterns. By applying this model, between people differences in within people change overtime was captured (Jung & Wickrama, 2008). This means that subgroups of individuals were identified based on trajectories of change of the shattered safety correlates. The slope factor loadings for the observations at waves five through nine (ages 13–24) were set to 0, 0.16, 0.35, 0.63, and 1 to reflect the time between the waves. Data were fitted using both linear and quadratic growth models, to determine best fit. The means and variances of the slope and intercept factors were initially estimated without constraints. Constraints were introduced as needed to ensure model convergence.

GMMs were estimated for one-, two-, three-, four-, and five- class models (up until there was no further improvement) to determine which solutions best captured the heterogeneity in the data. The best model fit was chosen based on interpretability, parsimony, high entropy (near 1), and model-fit criteria, such as the smallest AIC and adjusted-BIC values (lower indicates better fit). Vuong-Lo-Mendell-Rubin Likelihood Ratio tests (VLMR LRT) were conducted to test to what extent each additional class significantly improved model fit. To determine to what extent the linear or quadratic growth model fit the data better, the Root Mean Square Error Approximation (RMSEA; close fit </= 0.05), Comparative Fit Index (CFI; good fit >/= 0.95), Tucker-Lewis Index (TLI; good fit >/= 0.95), and Standardized Root Mean Square Residual (SRMR; good fit: </= 0.08) were also consulted for the one-class model. Linear and linear+quadratic growth model information will be provided.

To compare the trajectory groups of the best model fit in terms of sociodemographic factors (sex, SES, higher education level, and cultural background), the Bolck-Croon-Hagenaar (BCH) procedure was used (Asparouhov & Muthén, 2019). This method is recommended because it accounts for the probabilistic nature of class membership resulting in more unbiased estimates compared to other approaches (Bakk & Vermunt, 2016). Finally, the same BCH procedure was applied to estimate the relationship between the trajectory groups and the distal outcomes (conspiracy mentality and violent extremist attitudes).

### Sensitivity Analysis

The measurement of bullying victimization changed in wave nine and one original item that was assessed in previous waves was also discontinued. To test robustness of results, GMMs were reran using only three items that were consistently available for waves five through nine (for a full overview of items used per construct, see Table 1 in the Supplementary materials).

## Results

### Descriptives

Correlations between different shattered safety correlates (bullying victimization, distress, distrust) were overall small (see Table 1). Correlations between the same shattered safety correlates across waves ranged from small to moderate.

**Table 1:** Pearson correlation matrix of shattered safety correlates, conspiracy mentality and violent extremist attitudes . Means (se) z-scores, and omega's of continuous scales (*N* = 1482)

	1	2	3	4	5	6	7	8	9	10	11	12	13	14	15	16	17	M (se)	ω
1. W9 Cons	-																	2.58 (0.02)	0.859
2. W9 VE	0.066*	-																1.58 (0.02)	0.852
3. W5_BullV	0.029	0.037	-															1.70 (0.02)	0.785
4. W6_BullV	− 0.027	0.078	0.477***	-														1.64 (0.02)	0.726
5. W7_BullV	− 0.013	− 0.002	0.365***	0.470***	-													1.45 (0.02)	0.739
6. W8_BullV	0.003	0.072*	0.284***	0.304***	0.420***	-												1.40 (0.01)	0.674
7. W9_BullV	0.060	0.112**	0.206***	0.262***	0.316***	0.400***	-											1.38 (0.02)	0.741
8. W5_Distress	− 0.033	− 0.019	0.403***	0.257***	0.206***	0.152***	0.171***	-										2.19 (0.02)	0.833
9. W6_Distress	0.004	− 0.050	0.221***	0.283***	0.221***	0.194***	0.156***	0.545***	-									2.33 (0.02)	0.849
10. W7_Distress	0.099*	− 0.103*	0.183***	0.203***	0.248***	0.233***	0.197***	0.461***	0.614***	-								2.41 (0.02)	0.858
11. W8_Distress	0.047	0.043	0.144***	0.157***	0.189***	0.323***	0.240***	0.351***	0.477***	0.586***	-							2.40 (0.02)	0.874
12. W9_Distress	0.008	0.073	0.104***	0.145***	0.151***	0.162***	0.285***	0.311***	0.411***	0.487***	0.577***	-						2.48 (0.02)	0.864
13. W5_Distrust	− 0.035	0.045	0.121***	0.079**	0.093***	0.071*	0.032	0.175***	0.154***	0.155***	0.152***	0.121***	-					2.39 (0.02)	0.738
14. W6_Distrust	− 0.072*	0.058	0.121***	0.103***	0.041	0.086**	0.058	0.172***	0.244***	0.212***	0.186***	0.172***	0.392***	-				2.59 (0.02)	0.776
15. W7_Distrust	0.068	0.035	0.036	0.032	0.074**	0.067*	0.101***	0.077*	0.149***	0.219***	0.167***	0.160***	0.290***	0.511***	-			2.68 (0.02)	0.827
16. W8_Distrust	0.146***	− 0.031	0.013	− 0.026	− 0.023	0.092**	0.068*	0.014	0.076**	0.133***	0.215***	0.189***	0.192***	0.366***	0.503***	-		2.66 (0.02)	0.843
17. W9_Distrust	0.308***	− 0.029	0.003	0.045	0.006	0.064*	0.165***	0.013	0.084**	0.132***	0.220***	0.271***	0.170***	0.340***	0.413***	.581***	-	2.61 (0.02)	0.863

Notes. Mean and Standard Errors values from raw variable scores. W5-9 = waves, Cons = Conspiracy mentality, VE = Violent extremist attitudes. BullV =distress (anxiety and depression), Distrust = general sense of distrust.Correlations in bold are significant*p< .05; ** p< .01; *** p< .001

### Growth Mixture Model Results

Overall, model fit statistics (AIC, BIC, adj-BIC, RMSEA, CFI, TLI, and SRMR) indicated that the linear-quadratic model better fit the data for the one-class model, compared to the linear-model (see Table 2). A similar pattern was observed for the two-to-five class models. Table 3 presents a summary of the test statistic for the linear model and Table 4 for the linear-quadratic model. Due to its better fit, only the linear-quadratic model fit criteria results are discussed here. Considering the VLMR LRT outcomes, the three- and five class-model provided the best fit to the data. Of these two, the three class-model was selected, while the five-class model had slightly lower AIC, BIC, and Adj.-Bic values, the entropy value indicated poorer class separation, not justifying the complexity of adding two small classes. Overall, the three-class model had good fit statistics, was well-fitting, and provided the most conceptually meaningful solution. For an overview of the alternative two, four, and five class models, see Table 2 in the Supplementary materials.

**Table 2:** Model Fit Information for the GMM Model for the shattered safety correlates

	AIC	BIC	Adj-Bic	RMSEA	CFI	TLI	SRMR
Linear	49762.525	49985.173	49851.752	0.049	0.927	0.918	0.039
Linear + quadratic^1^	49652.172	49970.241	49779.639	0.045	0.950	0.931	0.036

*Notes*. ^1^Variance for the slope of the latent factor distress was fixed to zero

**Table 3:** Model Fit Information GMM models for shattered safety correlates – linear growth models (*N* = 1482)

Number of Classes	AIC	Bic	Adj-Bic	Entropy	(VLMR LRT)		Class count (%)
					Value	*P*-Value	
2	49377.044	49636.800	49481.142	0.956	-24839.263	0.000	1: 1427 (96.3%); 2: 55 (3.7%),
3	49088.270	49385.134	49207.239	0.933	-24639.522	0.000	1: 1338 (90.3%); 2: 93 (6.3%); 3: 51 (3.4%)
4	49040.254	49342.420	49161.348	0.823	-24518.131	0.145	1: 1163 (78.5%); 2: 177 (11.9%); 3: 100 (6.8%); 4: 42 (2.8%)
5	48935.964	49275.238	49071.929	0.847	-24463.127	0.167	1: 1126 (76.0%); 2: 189 (12.8%); 3: 120 (8.1%); 4: 40 (2.7%); 5: 7 (0.5%)

*Notes*. Variance of the slope of the latent factor bullying victimization was fixed to zero for 4 and 5 class configurations

**Table 4:** Model Fit Information GMM models for shattered safety correlates – linear + quadratic growth models (*N* = 1482)

Number of Classes	AIC	Bic	Adjusted-BIC	Entropy	(VLMR LRT)		Class count (%)
					Value	*P*-Value	
2	49334.067	49705.148	49482.778	0.955	-24820.964	0.009	1: 1431 (96.6%); 2: 51 (3.4%)
3	48983.927	49408.019	49153.882	0.927	-24597.034	0.001	1: 1322 (89.2%), 2: 116 (7.8%), 3: 44 (3.0%).
4	48821.092	49298.196	49012.292	0.928	-24411.963	0.149	1: 1302 (87.9%), 2: 100 (6.7%), 3: 42 (2.8%), 4: 38 (2.6%).
5	48701.252	49231.367	48913.696	0.847	-24320.546	0.037	1: 1190 (80.3%), 2: 114 (7.7%), 3: 99 (6.7%), 4: 43 (2.9%), 5: 36 (2.4%).

*Notes*. Variance of the slope of the latent factor distress was fixed to zero for all class configurations

### Developmental Shattered Safety Trajectories

**Fig. 1:**
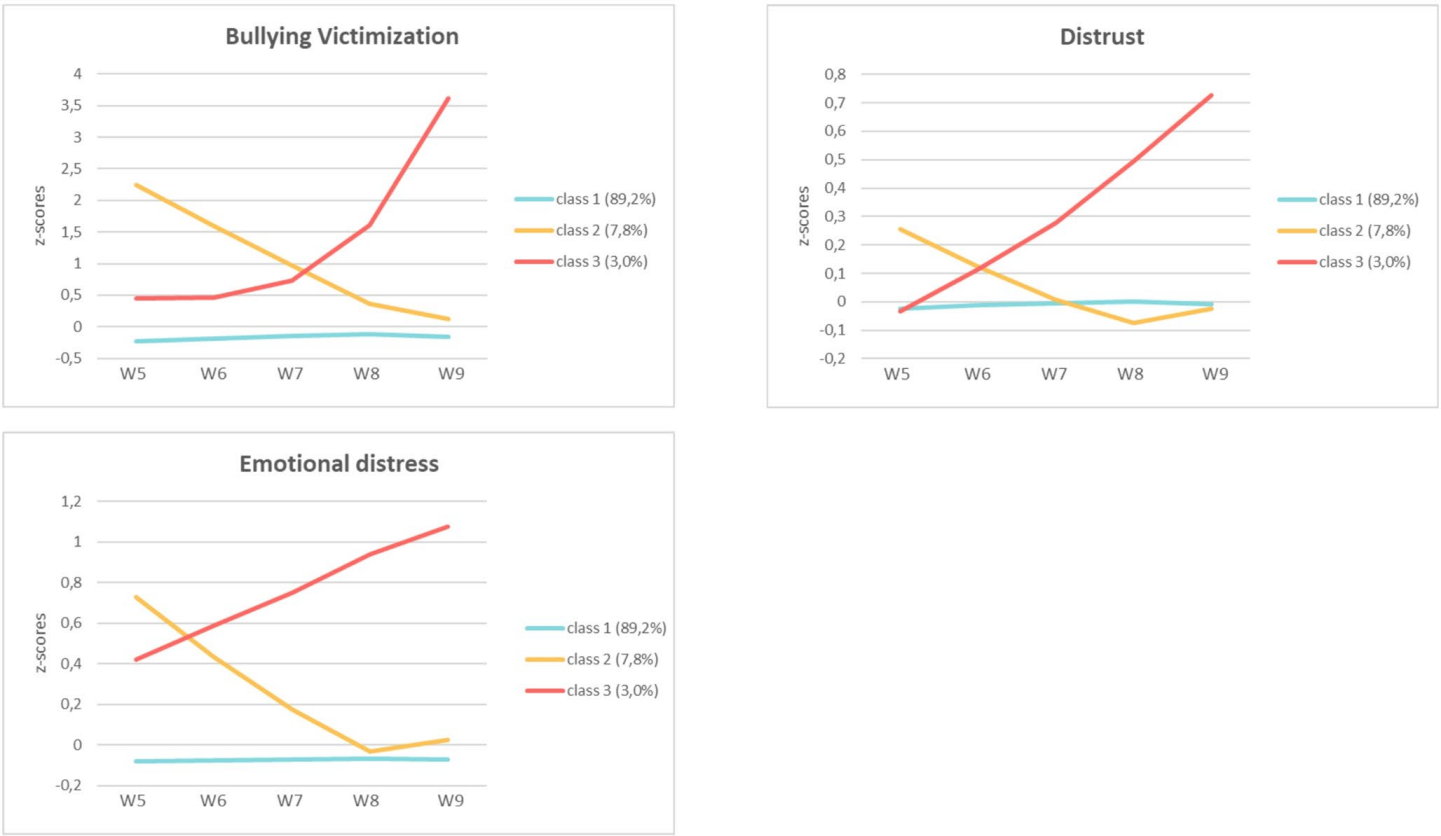
Trajectories for the chosen 3-class model (linear + quadratic GMM) representing the estimated z-scores means of shattered safety correlates scores per class across waves. Class 1 represents the “low stable” trajectory, class 2 the “decreasing” trajectory and class 3 the “increasing” trajectory. Note: Y-axis represents the z-scores and the X-axis represents the waves (W5 = age 13, W6 = age 15, W7 = age 17, W8 = age 20, W9 = age 24)

The intercept and slopes (linear and quadratic) for each trajectory group in the three-class model are presented in Table 5. Most adolescents (89.2%) belonged to the trajectory group which was named “low stable”. This trajectory group had relatively low starting levels of shattered safety correlates (bullying victimization, emotional distress, distrust) that remained stable and low overtime. A smaller proportion of participants (7.8%) initially demonstrated elevated levels of shattered safety correlates, in particular bullying victimization and distress, which gradually decreased overtime as they got older. Therefore, this group was named “decreasing.” Finally, for some young individuals (3.0%) in the “increasing” trajectory group, shattered safety correlates were initially average to moderate, but then increased overtime. Figure 1 provides a visual display of the developmental risk factor trajectories per group. Finally, sociodemographic characteristics were compared between trajectory groups (see Table 6), no differences were found except for a lower percentage of males being present in the low stable group, compared to the increasing (χ² = 6.873, *p* = .009) and decreasing (χ² = 10.082, *p* = .001) trajectory groups.

**Table 5:** Growth parameters for chosen linear + quadratic 3-class model (standardized results)

	Factor	Bullying Victimization			Distress			Distrust		
	Parameter	Intercept (SE)	Linear (SE)	Quadratic (SE)	Intercept (SE)	Linear (SE)	Quadratic (SE)	Intercept (SE)	Linear (SE)	Quadratic (SE)
Increasing (*n* = 44; 3.0%)	Standardized estimate	1.143 (0.539)	-0.259 (0.790)	2.218 (0.844)	0.577 (0.233)	*	-0.584 (0.851)	-0.048 (0.229)	0.428 (0.316)	-0.104 (0.323)
Decreasing (*n* = 116; 7.8%)	Standardized estimate	5.774 (0.678)	-2.662 (0.569)	1.444 (0.470)	1.000 (0.182)	*	1.755 (0.454)	0.370 (0.184)	-0.416 (0.197)	0.340 (0.204)
Low stable (*n* = 1322; 89.2%)	Standardized estimate	-0.600 (0.126)	0.218 (0.092)	-0.183 (0.077)	-0.112 (0.040)	*	-0.041 (0.130)	-0.033 (0.040)	0.033 (0.052)	-0.032 (0.053)

*Notes*. *Variance of the slope of the latent factor distress was fixed to zero

**Table 6:** Comparison of sociodemographic characteristics across trajectory groups

	Increasing (*n* = 44 )	Decreasing (*n* = 116)	Low stable (*n* = 1322)
Male	57.2%^a^	49.4%^a^	32.8%^b^
SES (mean)	45.44 ^a^	46.97 ^a^	45.88 ^a^
Higher education completed W9	45.5% ^a^	58.2% ^a^	54.4% ^a^
One or more parents born outside of Switzerland	85.9% ^a^	77.0% ^a^	76.0% ^a^

*Notes*. No significant difference was found between trajectory groups on the distal outcome when superscripts (a, b) are the same. A different superscript indicates trajectory group differ significantly on the outcome (*p* < .01)

### Distal Outcomes

Distal outcomes were compared between the trajectory groups and findings of the equality tests of means are summarized in Table 7. Using the BCH procedure, the trajectory groups differed significantly in conspiracy mentality level at age 24 (χ² (2) = 10.027, *p* = .007). Specifically, the increasing trajectory group had the highest level of conspiracy mentality, compared to the “low stable” (χ² = 9.753, *p* = .002) and “decreasing” trajectory (χ² = 8.382, *p* = .004) groups. The “low stable” and “decreasing” groups did not differ on conspiracy mentality (*p* = .270). For the distal outcome of violent extremist attitudes at age 24 an overall group difference was also found (χ² (2) = 9.871, *p* = .007). The “increasing” trajectory group had the highest level of violent extremist attitudes, compared to the “low stable” group (χ² = 9.093, *p* = .003). No other differences were found for violent extremist attitudes between trajectory groups.

**Table 7:** Comparison of distal outcomes z-scores across trajectory groups

	Conspiracy Mentality M (se)	Violent Extremist Attitudes M (se)
Increasing (*n* = 44)	0.542 (0.186)^a^	0.522 (0.180)^a^
Decreasing (*n* = 116)	-0.142 (0.114)^b^	0.113 (0.132)^a, b^
Low stable (*n* = 1322)	-0.009 (0.032)^b^	-0.033 (0.031)^b^

*Notes*. No significant difference was found between trajectory groups on the distal outcome when superscripts (a, b) are the same. A different superscript indicates trajectory group differ significantly on the outcome (*p* < .01)

### Sensitivity Analysis

Three similar trajectory groups – “increasing (3.0%),” “decreasing (9.0%),” “low stable (87.9%)” – were found when running the GMMs using only the three items for bullying victimization, see Tables 3, 4, 5 and 6 in the Supplementary materials for full results.

## Discussion

Understanding developmental pathways associated with conspiracy mentality and violent extremist attitudes is essential to help address and prevent these challenges in society. However, most studies that have jointly explored belief in conspiracy narratives and violent extremist attitudes have done so cross-sectionally (e.g., Duindam et al., 2025a; Rottweiler & Gill, 2020). To address this gap, the present study’s aim was to conduct GMMs on a large longitudinal z-proso sample, resulting in the identification of three trajectories based on shattered safety correlates (Shattered Assumption Theory; Janoff-Bullman, 1992) from young adolescence (13-years-old) to early adulthood (24-years-old). In line with the research hypothesis, most adolescents exhibited low levels of shattered safety at the start, which remained stable overtime; conform other research in community samples showing healthy functioning for most (Vella et al., 2019). Around 8% of adolescents were characterized by higher risks early on, which subsided overtime, comparable to the “low stable” majority group (i.e., adolescents without developmental risks).

For a small group of adolescents (3%), shattered safety was evident, reflected in a continued increase in its correlates–bullying victimization, emotional distress, and generalized distrust–across adolescence. Being bullied, combined with emotional distress (Reijntjes et al., 2010) and distrust beliefs (Tsomokos & Slavich, 2024), can have long-term mental health consequences. The findings highlight how these shattered safety correlates throughout adolescence are associated with distal outcomes: adolescents in the increasing problems group had the highest level of conspiracy mentality and violent extremist attitudes in young adulthood, compared to most peers (although no differences were found on violent extremist attitudescompared to the decreasing group). The results thereby provide initial evidence for the association between a conspirational and extremist worldview with developmental unsafety and insecurity, related to victimization, distress and distrust throughout adolescence. In terms of the sociodemographic characteristics of the trajectory groups, participants in the increasing and decreasing groups were more likely to be boys, in line with previous research demonstrating that males tend to have poorer mental health and engage in more at-risk behavior (Emmelkamp et al., 2020; Rice et al., 2021).

One of the main tasks during adolescence is to develop an understanding of oneself in relation to the world and others. Suffering and repeated harm, can disrupt basic safety and trust in the world and others (Shattered Assumption Theory; Janoff-Bulman, 1992). Current findings demonstrate how an increase in shattered safety from adolescence and into adulthood, is associated with the appeal of conspiracy narratives and violently extreme solutions in young adulthood. Whereas longitudinal studies with regards to conspiracy mentality, and to some extent violent extremist, are rare, the long term impact of wellbeing and mental health during childhood in other areas has previously been demonstrated (i.e., political voting; Girard & Okolikj, 2024, democratic citizenship; Okolikj & Girard, 2025, psychological distress; Dion et al., 2016).

The timing and duration of developmental problems may matter too, in terms of its impact on adulthood. Whereas early mental health problems can have an impact on adulthood more generally (Murray et al., 2022; Vergunst et al., 2023), the impact of adolescence onset problems may be more detrimental than childhood-limited issues (Bevilacqua et al., 2018). The 13-year-olds in the “decreasing” trajectory group initially had the highest levels of shattered safety risk, compared to the “increasing” trajectory group. However, developmental risks of adolescents in the latter group got worse, whereas the problems of those in the “decreasing” trajectory group went away. Previous research also found that adolescence-onset problems can have more impact in adulthood than problems limited to childhood (Bevilacqua et al., 2018). Another interpretation is that adolescence is a unique period of brain development during which neuronal synapses (i.e., connections between neurons) are eliminated partly in response to environmental experience. As a result of these processes, the adolescent brain may be especially sensitive to environmental stressors or negative experiences (Blakemore, 2019).

Alternatively, this finding could also hint at the importance of short-term processes on conspiracy mentality and violent extremist attitudes, over long-term processes. No differences were found in distal outcomes between the low stable majority and the decreasing problem subgroup. And while their developmental risk trajectories were different, the low stable and decreasing group both had average-to low shattered safety risks remaining at age 24. Whereas this was high for the increasing shattered safety trajectory group at age 24. Accordingly, there is evidence highlighting the importance of more proximal processes (e.g., media exposure, perceived threats, crises) on conspiracy mentality and violent extremist attitudes (den Elzen et al., 2026; van Prooijen & Douglas, 2017).

Interestingly, unlike findings from previous research (e.g., Katsantonis & Symonds, 2023; Vella et al., 2019), no “stable high” trajectory was identified among adolescents with sustaining difficulties. Perhaps z-proso participants are relatively well-functioning, having grown up in Zurich, one of the most affluent cities in the world (Ribeaud et al., 2022). The absence of a stable high trajectory group, could also be due to observed attrition pattern, which demonstrated somewhat higher dropout among those with more problem behaviors (Eisner et al., 2019; Ribeaud et al., 2022).

### Strengths, Limitations, and Future Directions

This is one of the first studies that examines to what extent shattered safety during adolescence is associated with conspiracy mentality and violent extremist attitudes in early adulthood. The z-proso project is one of the few longitudinal studies on a diverse, urban sample in Europe, with a focus on violence and delinquency (Ribeaud et al., 2022). For this study, five waves of shattered safety correlates were included, thereby covering almost the entire period from adolescence into young adulthood (13-to-24-years-old).

Despite the richness of these data, there are some limitations and important future directions to consider. One limitation is that conspiracy mentality and violent extremist attitudes were only included as distal outcomes. While violent extremist attitudes have been assessed in the z-proso study since age 17, conspiracy mentality was only added at age 24. For future research it would be crucial to assess how these challenges develop overtime and to what extent shattered safety correlates contribute to their progression. This could also provide more insight into the directionality of these relationships, as it is likely that shattered safety correlates and distal outcomes interact. For example, developing a conspiracy mentality is likely not only influenced by emotional distress. Having a conspiracy mentality has also been found to, in turn, exacerbate emotional distress (e.g., Liekefett et al., 2023).

Another limitation is that measures of online engagement (e.g., social media use, exposure to extremist content online) were not included in the present research even though the study period (2004–2022) captures a time marked by significant changes to the online world, including the rise of social media. Online experiences, such as the exposure to extremist content online, can have an impact on processes such as violent extremism (e.g., Pauwels & Schils, 2016) and conspiracy belief, although these relationships are often contingent on personal propensities and behavior (Enders et al., 2023). Future research should include online engagement measures, and zoom into the interplay with individual characteristics, to get more clarity on developmental processes leading to violent extremist attitudes and conspiracy belief both on- and offline.

While gendered pathways to violent extremist attitudes might have been operating, as male participants were overrepresented in the smaller trajectory groups, these could not be tested in the present study due to small cell sizes. Research on gendered pathways suggests that for some young men, aggrieved entitlement might play a role in the development of violent extremist attitudes, that is the conviction that one is unjustly denied privilege, status, or respect that “belongs” to men. This sense of aggrieved entitlement may have been fostered by personal grievance or victimization, and can result in an increased susceptibility to extremist narratives that promise a restoration of experienced status or power loss (Kimmel, 2018; Roose et al., 2022). The pattern of growing distress and distrust among the majority male participants in the increasing trajectory group, resonates with this research showing that perceived status threat can drive young men towards movement that promise restoration of their position through extreme means. The importance of gendered pathways has been stressed before (DCAF et al., 2019), and future research with larger samples should explore whether pathways from victimization to extremist attitudes operate differently for males and female adolescents.

In general, causal inference of shattered safety correlates on the distal outcomes cannot be assumed based on this study’s findings. One key question that arises is to what extent it is the trajectory over time, rather than the final endpoint of shattered safety correlates, that drives the differences in early adulthood conspiracy mentality and violent extremist attitudes. Except for general distrust and conspiracy mentality, no strong correlations were found in the final wave between shattered safety correlates and distal outcomes, perhaps pointing towards the relative importance of trajectories. Future research could benefit from Structural Equation Modeling (SEM) to disentangle how trajectories and endpoints relate to distal outcomes, while mediation analyses could additionally uncover mechanism of change, clarifying the impact of intermediate versus final risk. These type of analyses could also help to further disentangle why shattered safety could impact conspiracy mentality and violent extremism, as currently many views exist (e.g., Agnew, 2010; Green & Douglas, 2018).

Another limitation is the measurement of bullying victimization, as the assessment changed in wave nine, and one original item assessed in previous waves was also discontinued. To ensure consistency in results, GMMs were reran using only the three items that were consistently available for waves five through nine. As trajectory groups were similar , the results based on all the available information (4 items for waves fives through eight, 3 items for wave nine) were reported.

In general, caution is warranted with regards to the selected shattered safety correlates. The current study adopted a developmental framework, driven by an interest in the trajectories of changes throughout adolescence. Some other relevant variables for shattered safety (e.g., negative life events, serious victimisation; z proso Project Team, 2024) were not included due to their static nature, meaning they were only measured at one or during few waves, which prevented the joint examination with the other developmental correlates overtime. Within the ecological-transactional models on child development and the impact of child maltreatment, a more long-term and process oriented view has been advocated for as psychopathology develops over time and is not due to static events or experiences (Cicchetti & Rizley, 1981; Sameroff & Mackenzie, 2003). Accordingly, shattered safety trajectories were examined based on longitudinally assessed developmental correlates. Future research on developmental pathways of conspiracy and violent extremist attitudes could expand on this work by including longitudinal assessment of other aspects of shattered safety.

Another limitation is warranted with regards to the attrition rates in the z-proso dataset. While over 70% of the sample at age 7 was still participating at age 20, an investigation of the non-monotonic attrition (i.e., participant may miss one wave, and then return to the study at a later stage) during the first ten years of the study, demonstrated that attrition was higher among those with low education, a migration background, and those with higher levels of problem behavior (Eisner et al., 2019; Ribeaud et al., 2022). This is important to consider when interpreting current findings, specifically as these factors have all been associated with conspiracy mentality and violent extremist attitudes previously (Emmelkamp et al., 2020; Hornsey et al., 2022). This means that the measured levels of this study’s constructs may have been an underestimate, as more at-risk participants may have dropped out. The relatively small (< 5%) “increasing” trajectory group may also be an underestimate. With regards to the two smaller trajectory groups more generally, parameter estimates were less precise, as indicated by the relatively larger standard error. Therefore, these findings should be interpreted with caution, although classification quality indices indicated good subgroup separation overall (entropy = 0.927, average posterior probabilities > 0.7 for 97.1% of the participants).

Furthermore, this study was done in a large, diverse, urban, sample, findings cannot be generalized beyond this context. It is important that current findings are replicated in different types of samples, especially also in more at-risk samples with higher levels of conspiracy mentality and violent extremist attitudes. Importantly, no stable high group was found of adolescents who had consistently high levels of shattered safety correlates, which might be the case in samples facing more adversity. As is often the case in general population samples, levels of problem behaviors are relatively low, compared to clinical samples, more research on the topic should be conducted in samples with higher violent extremist and conspirational attitudes.

In that context it is also important to mention that the presence of violent extremist attitudes with or without a conspiracy mentality, rarely translate into violent extremist behaviors (Kruglanski et al., 2022; Moskalenko & McCauley, 2021). For the prevention of violent extremist behaviors, often the goal of many governments and interventions, it is therefore essential that more research includes violent extremist behavior outcomes. In studying conspiracy mentality and violent extremism, it is also important to broaden the scope of risk factor domains assessed beyond the individual, to include the broader context in which the child is growing up. This includes, family functioning, parental wellbeing, and beyond extending to meso- (e.g., community influences) and macro-level (e.g., political conditions) factors, which can ultimately help understand why conspirational narratives might resonate, and even justify violence, for some people (Hornsey et al., 2022).

Finally, there is a need for an integrated theoretical framework that explains how conspiracy mentality – with or without violent extremist ideation – might develop. There are worries about new forms of violent extremism that do not neatly fit into specific religious or political ideologies, but are influenced by conspiracy belief and a variety of (only loosely connected) ideologies (Basit, 2021; Gartenstein-Ross et al., 2023). Some recent examples of conspiracy-inspired violent extremism include the QAnon-related storming of the US capitol and 5G towers being lit on fire under the influence of COVID-19 conspiracy narratives. Conspiracy belief and violent extremism have mostly been studied separately, and while most conspiracy believers will never become violent (Moskalenko & McCauley, 2021), their occasional co-occurrence warrant future joint examination (Joffe-Block, 2024; Powers, 2019; Satariano & Alba, 2020). However, theoretical guidance on the relation between the two is lacking, and the few models that do exist do not adopt a lifespan developmental focus (e.g., 3 N Model of Radicalization; Kruglanski et al., 2022). As overarching theoretical framework, the current study therefore builds on the Shattered Assumption Theory – a psychological model originally developed to describe consequences of traumatic experiences – because it illustrates how individual-level risk factors throughout one’s development may negatively impact macro-level belief systems, relevant for the adoption of conspiracy and violent extremist narratives. The selection of shattered safety correlates (bullying victimization, emotional distress, and general distrust) was further guided by several theoretical models on conspiracy mentality in adulthood (Existential Threat Model, Van Prooijen, 2020; Need-based Framework, Douglas et al., 2017) and the development of violent extremist attitudes more generally (General Strain Theory, Agnew, 2010; Social Control Theory; Gottfredson & Hirschi, 1990). However, the current correlate selection is not all inclusive, and future research should consider additional factors. To guide this work, the field would benefit from the development of an integrative theoretical framework to explain the emergence of conspiracy mentality and, for some, its link to violent extremism, while accounting for adolescents’ susceptibility and online influences.

### Implications

Conspiracy belief and violent extremism are pressing challenges frequently cited by governments and national security agencies as they seek to develop effective strategies (e.g., AIVD, 2023; Radicalisation Awareness Network, 2021). In recent years, more research has become available on the relationship between conspiracy belief and violent extremism (e.g., Basit, 2021; Moskalenko & McCauley, 2021; Rottweiler & Gill, 2020; Vegetti & Littvay, 2022), Most previous studies on this topic have focused on adults. This paper is among the first to highlight how developmental issues concerning an increasing shattered safety throughout adolescence might, for a small group, be associated with a conspiracy mentality and violent extremist attitudes later on. Whereas many interventions exist against violent extremism, few have been systematically evaluated, and most seem to focus on reducing bias and strengthening more flexible thinking styles (Jugl et al., 2021). While this may be important, current findings underscore the importance of adolescents’ emotional wellbeing and healthy peer dynamics more broadly. Moreover, they draw attention to the significance of considering developmental pathways of these challenges (Hornsey et al., 2022).

Specifically, the current study highlights the importance of safety during adolescent development, demonstrating the relevance of applying a public health-oriented perspective to these challenges (Miconi et al., 2021). Most adolescents in this study’s sample are doing well with low levels of shattered safety correlates, while only a small group (3%) developed increasing challenges associated with higher conspiracy mentality and violent extremist attitudes at age 24. For those adolescents in particular, policies are needed that foster socioemotional needs and safety, to provide them with resources that allow them to channel and challenge narratives that idealize violence (Stephens & Sieckelinck, 2020). To identify the small group who might be in need, regular mental health screening of wellbeing as a form of primary prevention could be considered. The importance of mental health monitoring and programming during adolescence has been recognized previously as this is an important and vulnerable phase during which most mental health disorders surface (while often remaining undiscovered until adulthood; Sacco et al., 2024). While schools are well-positioned to carry out such mental health screening, they often lack resources and training – with educational investments even declining further across Europe (European Commission: Directorate-General for Education, Youth, 2024). Furthermore, there has been a call for more developmentally sensitive mental health approaches geared towards young people (McGorry et al., 2024).

When concerns are flagged during screening of adolescents, several forms of secondary prevention and intervention are available for addressing shattered safety developmental correlates. With regards to bullying victimization, a topic that has received more attention lately, research has demonstrated that fostering a positive school climate is important for mitigating negative consequences of bullying (Zacharia & Yablon, 2022). In terms of addressing adolescents’ emotional distress, meta-analytical research has demonstrated that school-based intervention programs can be effective on average (van Loon et al., 2020), and cognitive behavioral therapies also have a strong evidence base for addressing emotional distress that reaches the disorder threshold (Schaeuffele et al., 2024). Beyond the school climate, the family environment is also important to consider with regards to addressing shattered safety correlates. A meta-analysis of samples spanning 22 countries, has indicated that factors such a good relationship with parents and general (financial) family wellbeing are associated with higher trust later in life, whereas abuse during childhood is associated with lower levels of trust perceptions (Kim et al., 2025).

In terms of interventions to address conspiracy mentality and violent extremist attitudes in adulthood, present findings highlight that levels of shattered safety developmental correlates were relatively higher at age 24 for the increasing trajectory group. This suggests that attention should be paid to adults’ wellbeing with regards to their sense of safety, although the results of effectiveness studies on interventions that aim to address the psychological needs that might trigger conspiracy belief have been unconvincing (Hornsey et al., 2022). Despite the developmental orientation of this paper, adult’ mental wellbeing should not be disregarded. Previous research found the highest support for violent extremism in those who simultaneously reported high conspiracy belief with psychological distress levels above a clinical cut off (Levinsson et al., 2022). Similarly, the majority of offenders convicted for QAnon related violent extremism reported mental health concerns (61.4%), which is a stark contrast from mental health issues among non-conspiracy related extremism (11.7%; Jensen & Kane, 2021).

## Conclusion

There is a lack of longitudinal research examining developmental pathways to conspiracy mentality and violent extremist attitudes. Therefore, this study aimed to examine one potential pathway involving shattered safety during adolescence. Three developmental trajectories (13-to-24-years-old) of shattered safety were identified based on bullying victimization, emotional distress and distrust, with most participants exhibiting low (89%) or subsiding (8%) levels of issues. For a small group of participants (3%) shattered safety increased throughout their adolescence, which was related to higher conspiracy mentality and violent extremist attitudes in young adulthood. These findings call for the adoption of a public health focus that prioritizes developmental wellbeing in preventing conspiracy mentality and violent extremist attitudes. This is in contrast with the (reactive and often counterproductive) security-driven approaches that have sometimes been adopted by states. Multileveled policy approaches are needed that protect democratic institutions and prevent violent extremist acts, while also giving significant attention to the strengthening of societal resilience through adolescent safety and wellbeing.

## Supplementary Information

Below is the link to the electronic supplementary material.

Supplementary Material 1

## Data Availability

The datasets generated and/or analyzed during the current study are not publicly available but are available from the corresponding author on reasonable request. The z-proso data are accessible via the SWISSUbase repository (Eisner et al., 2025; 10.48573/y4b2-b002).
