# Supplementary material for: Shattered Safety? Developmental Trajectories for Conspiracy Mentality and Violent Extremist Attitudes
Source: J Youth Adolesc. 2026 Apr 29;55(7):1659–77. doi: 10.1007/s10964-026-02350-9 (PMC13328236; doi:10.1007/s10964-026-02350-9)
Supplement: Supplementary file 1 — Supplementary Material 1 [file 10964_2026_2350_MOESM1_ESM.docx]

**Authors**: Hanne M. Duindam^1,6^, Lucia Hernandez-Pena^2,3^, Jessica J. Asscher^1^, Denis Ribeaud^4^, Manuel Eisner^4,5^

**Affiliations**: ^1^Clinical Child, Family and Education Studies, Faculty of Social Sciences, Utrecht University, Heidelberglaan 1, 3582 CE Utrecht, the Netherlands; ^2^Department of Psychiatry, Psychotherapy and Psychosomatics, Faculty of Medicine, RWTH Aachen, Pauwelsstrasse 30, 52074 Aachen, Germany; ^3^Department of Psychology, University of Cambridge, Downing PI, Cambridge CB2 3EB, United Kingdom; ^4^Jacobs Centre for Productive Youth Development, Universität Zürich, Andreasstrasse 15, CH-8050 Zurich, Switzerland; ^5^Institute of Criminology, University of Cambridge, Sidgwick Ave, Cambridge CB3 9DA, United Kingdom. ^6^Darwin College, University of Cambridge, Silver Str, Cambridge CB3 9EU, United Kingdom.

**Authors’ note**: All correspondence should be addressed to: Hanne M. Duindam, Faculty of Social Sciences, Utrecht University, [h.m.duindam@uu.nl](mailto:h.m.duindam@uu.nl).

| **Table 1. Items shattered safety constructs** | | | | |
| --- | --- | --- | --- | --- |
|  | Item no. | Text | Response scale | Wave |
| **Bullying victimization** |  | *Since [last year], how many times have other kids/adolescents/people…* | Never – almost daily (6) |  |
|  | 1 | …purposely ignored you or excluded you from something?* |  | 5-9 |
|  | 2 | …laughed at you, mocked you, or insulted you?* |  | 5-9 |
|  | 3 | …hit you, bitten you, kicked you, or pulled your hair?* |  | 5-9 |
|  | 4 | …purposely stolen, broken, or hidden your things?* |  | 5-8 |
|  |  |  |  |  |
| **Emotional distress** |  | *Please indicate below how you have felt in the last month. Please mark with*  *a cross whether you felt like the statements below either never, rarely,*  *sometimes, often or very often.* | Never-very often (5) | 5-9 |
|  | 1 | I was bored. |  |  |
|  | 2 | I cried. |  |  |
|  | 3 | I was anxious for no reason. |  |  |
|  | 4 | I felt unhappy. |  |  |
|  | 5 | I felt alone. |  |  |
|  | 6 | I couldn’t fall asleep at night. |  |  |
|  | 7 | I was sad without knowing why. |  |  |
|  | 8 | I was worried. |  |  |
|  | 9 | I harmed myself on purpose (e.g. cut my arm, tore open wounds, hit my head, tore out my hair). |  |  |
|  |  |  |  |  |
| **General distrust** |  | *This part is about how you see other people in everyday life. Below are various statements. Please mark with a cross how true these statements are for you.* | Fully untrue-fully true (4) | 5-9 |
| (reverse coded) | 1 | Most people can be trusted. |  |  |
|  | 2 | People usually try to help other people. |  |  |
|  | 3 | Most people try to be fair. |  |  |
|  |  |  |  |  |
| **Conspiracy mentality** |  |  | Fully untrue-fully true (4) | 9 |
|  | 1 | Many very important things happen in the world, which the public is never informed about |  |  |
|  | 2 | Politicians usually do not tell us the true motives for their decision. |  |  |
|  | 3 | Government agencies closely monitor all citizens. |  |  |
|  | 4 | Events which superficially seem to lack a connection are often the result of secret activities. |  |  |
|  | 5 | There are secret organisations that greatly influence political decisions. |  |  |
|  |  |  |  |  |
| **Violent extremist attitudes** |  |  | Fully untrue-fully true (4) | 7-9 |
|  |  | It’s sometimes necessary to use violence to fight against things that are very unjust |  |  |
|  |  | Sometimes people have to resort to violence to defend their values, convictions or religious beliefs |  |  |
|  |  | It’s OK to support groups that use violence to fight injustices |  |  |
|  |  | It is sometimes necessary to use violence, commit attacks or kidnap people to fight for a better world |  |  |
| *Items for bullying victimization used in the current study | | | | |

| **Table 2. Mean (SD) z-scores developmental factors for alternative profile solutions** | | | | | | | | | | | | | | | |
| --- | --- | --- | --- | --- | --- | --- | --- | --- | --- | --- | --- | --- | --- | --- | --- |
|  | W5-BulV | W6- BulV | W7- BulV | W8- BulV | W9- BulV | W5-Distress | W6- Distress | W7- Distress | W8- Distress | W9- Distress | W5-Distrust | W6-Distrust | W7-Distrust | W8-Distrust | W9-Distrust |
| **2-class solution** | | | | | | | | | | | | | | | |
| Profile 1 (*n* = 1431) | -0.03 (0.97) | -0.03 (0.95) | -0.04 (0.95) | -0.07 (0.89) | -0.13 (0.71) | -0.02 (0.99) | -0.03 (0.99) | -0.03 (0.99) | -0.04 (0.98) | -0.04 (0.98) | 0.00 (1.00) | -0.01 (1.00) | -0.01 (1.00) | -0.01 (1.00) | -0.03 (1.00) |
| Profile 2 (*n* = 51) | 0.77 (1.40) | 0.96 (1.66) | 1.06 (1.68) | 1.75  (1.72) | 3.61 (0.96) | 0.58 (1.02) | 0.78 (1.09) | 0.74 (0.92) | 1.05 (1.00) | 1.01 (0.96) | -0.12  (0.94) | 0.26 (1.07) | 0.28 (0.88) | 0.29 (0.93) | 0.70 (0.86) |
| **4-class solution** | | | | | | | | | | | | | | | |
| Profile 1 (*n* = 42) | 0.44 (0.92) | 0.59 (1.27) | 0.68 (1.37) | 1.33 (1.52) | 3.68 (0.93) | 0.47 (0.92) | 0.68 (1.06) | 0.62 (0.92) | 0.92 (0.99) | 1.06 (0.96) | -0.20 (0.99) | 0.19 (1.12) | 0.22 (0.91) | 0.24 (0.97) | 0.73 (0.88) |
| Profile 2 (*n* = 38) | 0.75 (1.01) | 1.40 (1.71) | 3.19 (1.89) | 2.47 (1.97) | 0.37 (0.89) | 0.33 (0.93) | 0.63 (0.94) | 1.01 (1.20) | 1.03 (1.21) | 0.47 (1.09) | 0.03 (0.67) | 0.35 (0.87) | 0.33 (1.03) | 0.24 (0.90) | 0.16 (1.06) |
| Profile 3 (*n* = 100) | 2.53 (0.99) | 1.37 (1.42) | 0.67 (1.12) | 0.40 (1.13) | 0.07 (1.02) | 0.91 (1.18) | 0.36 (1.11) | 0.16 (1.01) | 0.11 (1.06) | 0.01 (0.95) | 0.33 (1.02) | 0.16 (1.04) | 0.00 (1.09) | -0.11 (1.04) | -0.08 (0.90) |
| Profile 4 (*n* = 1302) | -0.24 (0.65) | -0.17 (0.78) | -0.17 (0.71) | -0.15 (0.77) | -0.15 (0.70) | -0.10 (0.95) | -0.07 (0.97) | -0.06 (0.97) | -0.07 (0.96) | -0.05 (0.98) | -0.02 (1.00) | -0.03 (0.99) | -0.02 (0.99) | -0.01 (1.00) | -0.02 (1.00) |
| **5-class solution** | | | | | | | | | | | | | | | |
| Profile 1 (*n* = 1190) | -0.23 (0.65) | -0.17 (0.77) | -0.18 (0.71) | -0.18 (0.74) | -0.16 (0.69) | -0.11 (0.94) | -0.15 (0.92) | -0.23 (0.83) | -0.25 (0.78) | -0.14 (0.94) | -0.03 (0.99) | -0.06 (0.99) | -0.08 (0.99) | -0.08 (0.97) | -0.09 (0.99) |
| Profile 2 (*n* = 114) | -0.33 (0.64) | -0.08 (0.87) | -0.03 (0.78) | 0.08 (0.93) | -0.08 (0.74) | 0.05 (0.97) | 0.79 (1.11) | 1.49 (0.84) | 1.56 (0.83) | 0.80 (0.99) | 0.05 (1.08) | 0.29 (0.97) | 0.57 (0.85) | 0.67 (0.97) | 0.62 (0.84) |
| Profile 3 (*n* = 43) | 0.43 (0.91) | 0.58 (1.26) | 0.66 (1.36) | 1.35 (1.50) | 3.68 (0.93) | 0.50 (0.92) | 0.66 (1.05) | 0.64 (0.92) | 0.92 (0.97) | 1.06 (0.96) | -0.20 (0.98) | 0.20 (1.11) | 0.19 (0.93) | 0.29 (1.00) | 0.73 (0.88) |
| **Profile 4 (*n* = 36)** | 0.81 (1.00) | 1.41 (1.74) | 3.21 (1.95) | 2.63 (1.94) | 0.38 (0.88) | 0.31 (0.93) | 0.64 (0.94) | 1.00 (1.15) | 1.08 (1.21) | 0.45 (1.10) | 0.05 (0.68) | 0.37 (0.89) | 0.37 (1.03) | 0.26 (0.89) | 0.13 (1.01) |
| **Profile 5 (*n* = 99)** | 2.54 (0.99) | 1.38 (1.42) | 0.67 (1.12) | 0.42 (1.13) | 0.07 (1.03) | 0.93 (1.18) | 0.36 (1.11) | 0.16 (1.01) | 0.13 (1.05) | 0.02 (0.95) | 0.33 (1.02) | 0.18 (1.03) | 0.00 (1.09) | -0.11 (1.04) | -0.08 (0.90) |

| **Table 3. Model Fit Information for the LGC Model for shattered safety correlates – bullying victimization measured by 3 items each wave** | | | | | | | |
| --- | --- | --- | --- | --- | --- | --- | --- |
|  | **AIC** | **BIC** | **Adj-Bic** | **RMSEA** | **CFI** | **TLI** | **SRMR** |
| Linear | 49759.843 | 49982.491 | 49849.070 | 0.049 | 0.929 | 0.920 | 0.039 |
| Linear + quadratic* | 49702.439 | 50020.507 | 49829.905 | 0.049 | 0.941 | 0.917 | 0.033 |

*Notes*. *Variance of the slope of the latent factor distress was fixed to zero.

| **Table 4. Model Fit Information GMM models for shattered safety correlates – bullying victimization measured by 3 items each wave – linear growth models (*N* = 1482)** | | | | | | | | |
| --- | --- | --- | --- | --- | --- | --- | --- | --- |
| **Number of Classes** | **AIC** | **Bic** | **Adj-Bic** | **Entropy** | **VUONG-LO-MENDELL-RUBIN LRT TEST** | | **Class count (%)** |  |
|  |  |  |  |  | Value | P-Value |  |  |
| 2 | 49386.003 | 49645.759 | 49490.101 | 0.955 | -24837.921 | 0.000 | 1: 1428 (96.4%); 2: 54 (3.6%) |  |
| 3 | 49129.322 | 49426.186 | 49248.291 | 0.926 | -24644.001 | 0.004 | 1: 1345 (90.1%); 2: 90 (6.7%); 3: 47 (3.2%) |  |
| 4* | 49074.379 | 49376.545 | 49195.473 | 0.821 | -24541.159 | 0.2740 | 1: 1154 (77.9%); 2: 189 (12.8%); 3: 99 (6.7%); 4: 40 (2.7%) |  |

*Notes*. *Variance of the slope of the latent factor bullying victimization was fixed to zero for this class configuration.

| **Table 5. Model Fit Information GMM models for shattered safety correlates – bullying victimization measured by 3 items each wave – linear+quadratic growth models (*N* = 1482)** | | | | | | | | |
| --- | --- | --- | --- | --- | --- | --- | --- | --- |
| **Number of Classes** | **AIC** | **Bic** | **Adj-Bic** | **Entropy** | **VUONG-LO-MENDELL-RUBIN LRT TEST** | | **Class count (%)** |  |
|  |  |  |  |  | Value | P-Value |  |  |
| 2 | 49336.030 | 49707.110 | 49484.110 | 0.995 | -24819.575 | 0.012 | 1: 1431 (96.6%); 2: 51 (3.4%) |  |
| 3 | 49003.237 | 49427.329 | 49173.192 | 0.920 | -24598.015 | 0.000 | 1: 1303 (87.9%); 2: 134 (9.0%); 3: 45 (3.0%) |  |
| 4* | 48811.167 | 49245.861 | 48985.372 | 0.922 | -24450.717 | 0.1243 | 1: 1281 (86.4%); 2: 101 (6.8%); 3: 65 (4.4%); 4: 35 (2.4%) |  |

*Notes*. *Variance of the linear slope of the latent factor distress was fixed to zero for all class configurations, for the 4 classes configuration residual variance of the quadratic slope of the latent factor bullying victimization was also fixed to zero.

| **Table 6. Growth parameters for linear + quadratic 3-class model (standardized STDYX results) – bullying victimization measured by 3 items each wave** | | | | | | | | | | |
| --- | --- | --- | --- | --- | --- | --- | --- | --- | --- | --- |
|  | **Factor** | **Bullying Victimization** | | | **Distress** | | | **Distrust** | | |
|  | **Parameter** | **Intercept (SE)** | **Linear (SE)** | **Quadratic (SE)** | **Intercept (SE)** | **Linear (SE)** | **Quadratic (SE)** | **Intercept (SE)** | **Linear (SE)** | **Quadratic (SE)** |
| Increasing (*n* = 45; 3.0%) | Standardized estimate | 1.464 (0.737) | -0.418 (0.630) | 2.393 (0.717) | 0.638 (0.236) | * | -0.571 (0.745) | -0.071 (0.239) | 0.463 (0.323) | -0.140 (0.332) |
| Decreasing (*n* = 134; 9.0%) | Standardized estimate | 5.532 (0.729) | -3.276 (0.800) | 1.903 (0.555) | 0.938 (0.161) | * | 1.985 (0.499) | 0.392 (0.144) | -0.451 (0.204) | 0.378 (0.209) |
| Low stable (*n* = 1303; 87.9%) | Standardized estimate | -0.670 (0.153) | 0.323 (0.110) | -0.257 (0.086) | -0.122 (0.041) | * | -0.093 (0.131) | -0.040 (0.041) | 0.041 (0.052) | -0.039 (0.054) |
| *Variance of the linear slope of the latent factor distress was fixed to zero | | | | | | | | | | |
